# The complete genome sequence of *Pantoea agglomerans* NBBC-01, isolated from rot potato tubers

**DOI:** 10.1128/MRA.00748-23

**Published:** 2023-10-17

**Authors:** Yueying Wang, Ling Chen, Fang Liu, Zhigang Zhang, Fei Zhang, Kaimei Wang, Wei Fang

**Affiliations:** 1 National Biopesticide Engineering Technology Research Centre, Hubei Biopesticide Engineering Research Centre, Hubei Academy of Agricultural Sciences, Biopesticide Branch of Hubei Innovation Centre of Agricultural Science and Technology, Wuhan, China; University of Arizona, Tucson, Arizona, USA

**Keywords:** *Pantoea agglomerans*, genome sequence, rot potatoes, plant-parasitic nematode

## Abstract

The Gram-negative bacterium *Pantoea agglomerans* has been isolated from various habitats including disease plants. Here, we present the genome of *P. agglomerans* strain NBBC-01 obtained from rotten potatoes that were infected by *Ditylenchus desstructor*. The genome information will prove advantageous in elucidating its ecological role.

## ANNOUNCEMENT

*Pantoea agglomerans* has been shown to promote plant growth (1, 2) and cause plant diseases (3, 4). This study presents the genome of *P. agglomerans* strain NBBC-01. The data could offer novel insights into the interaction mechanisms of *P. agglomerans* with plants. NBBC-01 was isolated from rotten potatoes collected from a commercial potato field (30.72N, 114.02E) in Wuhan, China, which was infected with a plant-parasitic nematode *Ditylenchus destructor* (5). Each nematode wound was rinsed with 5-mL sterile water, and the water was collected as bacterial suspensions. The suspensions were cultivated on lysogeny broth (LB) agar plates and incubated at 30°C overnight. A single colony was chosen for genome sequencing.

The purified colony was inoculated into the LB medium and incubated at 30°C until the mid-logarithmic growth phase. Genomic DNA samples for both long- and short-read sequencing were produced from the same genomic extraction using Blood & Cell Culture DNA Kits (Qiagen, Germany) following the manufacturer’s protocols. The DNA purity, concentration, and integrity were assessed with NanoDrop One spectrophotometer (NanoDrop Technologies, Wilmington, DE, USA), Qubit v.4.0 Fluorometer (Invitrogen, USA), and 0.75% agarose gel electrophoresis, respectively. Size selection (20–30 kb) was carried out for long-read sequencing utilizing the PippinHT system (Sage Science, USA). The DNA library was generated without DNA shearing utilizing a ligation sequencing kit [SQK-LSK-109; Oxford Nanopore Technologies, Ltd. (ONT]), Oxford, UK] and sequenced with a Nanopore PromethION sequencer instrument (ONT) on a R9.4.1 flow cell (FLO-MIN106). Guppy v.4.4.2 (6) was used to conduct base calling, barcode segmentation, and adapter sequence removal. Totally 119,589 reads (2,667  Mb) were produced, with an average length of 22,308  bp and an N50 of 30,727 bp. Genomic DNA was randomly fragmented by Covaris LE220 focused ultrasonicator (USA) and chosen by AMPure XP magnetic beads (Beckman, Germany) to an average size of 200–400 bp for short-read sequencing. MGIEasy FS PCR Free DNA library preparation set (MGI Tech Co., Ltd., China; cat no. 1000013455) was used to prepare the paired-end DNA library. The sequencing procedure was conducted using a DNBSEQ-T7 (MGI Tech Co., Ltd.), with reads of 150 nucleotides in length. The raw data underwent processing using the FASTQ preprocessing software fastp v.0.23.2 (7) in order to remove adapters and low-quality data. This resulted in a total of 7,940,828 short reads (1,191 Mb).

The high-quality short- and long-read sequences were assembled into a whole chromosome and plasmids using Unicycler v.0.4.9 (8). All software in this study used default parameters. The average coverage is 541.5× for ONT and 241.7× for MGI. The genome size is 4,927,193 bp, comprising a circular chromosome and five plasmids as indicated in Table 1. The genome sequence was annotated using National Center for Biotechnology Information PGAP v.5.3 (9) with default parameters. The genome comprises a total of 4,529 genes, consisting of 80 tRNAs and 22 rRNAs (Table 1). The average nucleotide identities analysis (10, 11) revealed that the genome of NBBC-01 showed the highest homology (98.65%) against *P. agglomerans* DSM3493^T^ (GenBank accession: GCA_900185875). The values of digital DNA–DNA hybridization (12, 13) between NBBC-01 and *P. agglomerans* NBRC 102470 (GenBank accession: GCA_001598475) is 88.7%. Thus, NBEC-018 was identified as a *P. agglomerans* isolate.

**TABLE 1:** Genome features of *Pantoea agglomerans* NBBC-01

Features	Length (bp)	G + C content (%)	No. of genes	No. of tRNAs	No. of rRNAs	Accession numbers
Chromosome	4,007,306	55.6	3,631	80	22	CP099728
Plasmid pNBBC01-1	531,583	53.55	508	0	0	CP099729
Plasmid pNBBC01-2	197,206	52.54	183	0	0	CP099730
Plasmid pNBBC01-3	81,391	55.03	87	0	0	CP099731
Plasmid pNBBC01-4	71,619	53.05	82	0	0	CP099732
Plasmid pNBBC01-5	38,088	44.91	38	0	0	CP099733
Total	4,927,193		4,529	80	22	

## Data Availability

The whole genome shotgun project's accession number is CP099728-CP099733, and it has been deposited in GenBank. Sequence Read Archive database accession numbers SRR19974010 (MGI) and SRR19974024 (ONT) were used to identify the raw sequencing data.
